# Epidemiology of impaction colic in donkeys in the UK

**DOI:** 10.1186/1746-6148-3-1

**Published:** 2007-02-02

**Authors:** Ruth Cox, Christopher J Proudman, Andrew F Trawford, Faith Burden, Gina L Pinchbeck

**Affiliations:** 1 Faculty of Veterinary Science, University of Liverpool, Leahurst, Neston, Wirral, CH64 7TE, UK; 2 The Donkey Sanctuary, Sidmouth, Devon, EX11 1DS, UK

## Abstract

**Background:**

Colic (abdominal pain) is a clinical condition of serious concern affecting the welfare and survival of donkeys at the Donkey Sanctuary in the UK. One of the most commonly reported causes is due to impacted ingesta in the large intestine ("impaction colic"). However little is known about the incidence of, or risk factors for, this condition. Here we describe the epidemiology of colic in donkeys, specifically impaction colic. We focus on temporal aspects of the disease and we identify environmental and management related risk factors for impaction colic in UK donkeys.

**Results:**

There were 807 colic episodes in the population of 4596 donkeys between January 1^st ^2000 and March 31^st ^2005. The majority (54.8%) of episodes were due to a suspected or confirmed diagnosis of impaction of the gastrointestinal tract. The mortality risk for all colics (51.1%) was higher than reported in other equids. The incidence rate of all colics (5.9 episodes per 100 donkeys per year) and of impaction colic (3.2 episodes) was similar to that in horses. A retrospective matched case-control study of all impaction colics from January 2003 (193) indicated that older donkeys, those fed extra rations and those that previously suffered colic were at increased risk of impaction. Lighter body weight, musculo-skeletal problems, farm and dental disease were also significantly associated with a diagnosis of impaction colic.

**Conclusion:**

To our knowledge this is the first study to estimate the incidence rate of colic in a large population of donkeys in the UK. In contrast to other equids, impaction was the most commonly reported cause of colic. We identified several risk factors for impaction colic. Increasing age, extra rations and previous colic are known risk factors for colic in other equids. Results support the hypothesis that dental disease is associated with impaction colic. Musculo-skeletal problems may be associated with colic for various reasons including change in amount of exercise or time at pasture. Other associated factors (weight and farm) are the subject of further research. Identification of risk factors for impaction colic may highlight high risk donkeys and may allow intervention strategies to be introduced to reduce the incidence of the disease.

## Background

The Donkey Sanctuary is a registered charity which aims to prevent the suffering of donkeys worldwide through the provision of professional advice, training and support on donkey welfare. The majority of the estimated 59 million donkeys worldwide are found in developing countries and are mostly used as draught or pack animals [1]. In the UK the Donkey Sanctuary cares for any donkey needing refuge including unwanted, rescued or sick donkeys. The Donkey Sanctuary farms in the south west of the UK have housed more than 11,000 individuals since 1968.

Management and medical details of all donkeys are recorded in the Donkey Sanctuary clinical database. Previous descriptive data from this database has shown that colic affects the health and survival of donkeys in this population. Furthermore impaction colic was implicated in more than half of the colic episodes seen in these donkeys between August 2000 and August 2001 and at least 45% did not recover from the disease [2,3].

In other equid populations, such as the horse, colic is also a significant problem in terms of morbidity, mortality and economics; in some equine populations it is the most common cause of death [4]. Epidemiological research has demonstrated that colic is complex and some studies have produced conflicting results about the impact of individual risk factors. However, many potential causal factors have been identified, for example, feed type, changes in management practices, lack of access to pasture or to water. While research has aimed to identify risk factors for colic in horses little is known about the incidence of the disease, or specific risk factors in donkeys which differ in many respects to other equids, for example in physiology, behaviour and management [5].

Here we describe the epidemiology of colic, in particular we describe temporal aspects of the disease, including incidence and seasonality and we also identify risk factors for the most common type of colic in donkeys at the Donkey Sanctuary.

## Results

### Incidence rate

The incidence rate for all types of colic was 5.9 new episodes per 100 donkeys per year. There were a total of 807 colic episodes in 694 (15.1%) individual donkeys in the population of 4596 donkeys that were resident at the Donkey Sanctuary at least some of the time during the study period (table 1). More than half (51.1%) the colic episodes resulted in mortality. Of these, 93% were euthanized, 4% were found dead and 3% died during treatment. When identifying cases of colic we included all that had been identified at post mortem as well as those identified during examination. Of the total of 412 colic cases that died, 159 were confirmed at post-mortem.

### Impaction colic

The majority (54.8%) of the colic cases were classified as impaction colic (table 1). Of the impaction colics, 51.4% did not survive the episode. The incidence rate of impaction colic was 3.2 episodes per 100 donkeys per year. Of the 227 donkeys that did not survive, 4 were found dead and the rest were euthanized – 93 on initial examination, 93 due to poor treatment response, 13 for other reasons (e.g. colitis, gut rupture) and 24 were unrecorded.

The site of 62.9% of impactions was identified – the majority of these (61.5%) were identified in the donkeys that did not survive the episode. The most common site was the pelvic flexure of the colon (39.6% of impactions were in this area) (table 2). However, the site of 37.1% of impactions was not identified – the majority of these (65.8%) occurred in the animals that survived the colic episode.

### Length of colic episode

The average length of all colic episodes from which donkeys recovered was 5.2 (SD 4.9) days, (n = 151, range = 1–31 days) (excluding surgical colics, or colics where the donkey died from other causes). The average length in donkeys diagnosed with impaction colic was 5.4 (SD 4.8) days (n = 110, range = 1–31 days).

There were 208 colic episodes where the recovery date of the colic was not specifically noted (excluding surgical colics). In these cases the colic episode lasted an average of at least 4.0 (SD 9.1) days, (i.e. the length of time between the start of the colic and the last date on which the colic was described in the examination notes). The average length of colic in donkeys diagnosed with impaction in this group was 5.9 (SD 12.8) days (n = 97, range = 1–104 days).

### Seasonality

**Figure 1:**
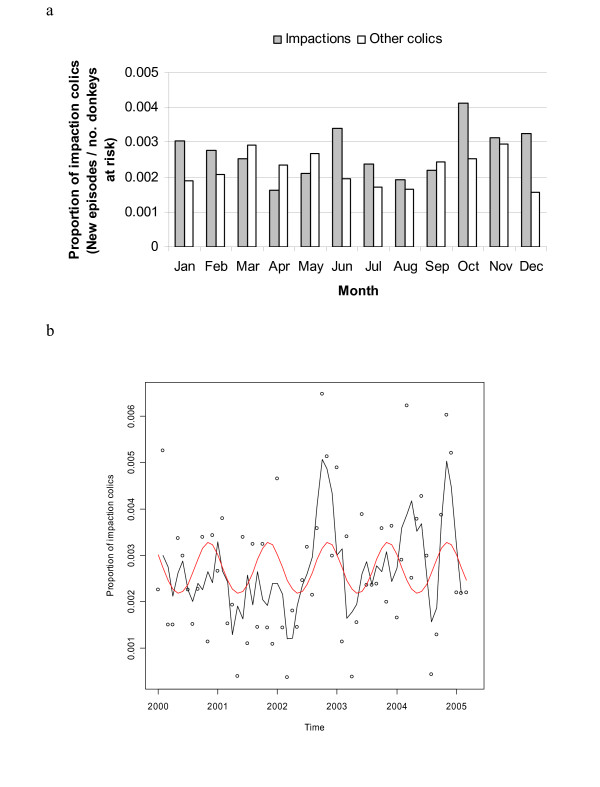
a – Proportion of all colics and impaction colics during each month of the year from January 2000 until March 2005. b – Time series analysis of the proportion of impaction colics during each month from January 2000 until March 2005. Plot shows proportion of impaction colics per month (open circles), fitted values from the regression model with 12 month cycle (red line) and raw data after smoothing with 3 month moving average (black line) which demonstrates a 12-month seasonal pattern in the occurrence of impaction colic with a peak in late autumn.

The binomial response regression model with a seasonal component demonstrated a 12 month seasonal pattern in the occurrence of impaction colic in this donkey population (table 3). Significant six monthly cycles were not identified. Although there were some departures in the raw data from the fitted values, peaks in the monthly prevalence of impactions tended to occur in late autumn and troughs in spring or summer (figure 1).

### Age

This is a population of old donkeys; in January 2005 the population (n = 2263) age was normally distributed with a mean of 25.2 (SD 8.5) years (range = 5 months-56 years). The mean age of donkeys that recovered from colic was 28.3(SD8.0)years and was significantly younger that those that died from colic, whose mean age was 30.9(SD 6.4)years, (P < 0.001).

### Dental disease

The database contained the dental charts of 2944 donkeys. A total of 34% (n = 1003) of the donkeys had missing teeth and altogether 43.6% (1284) of donkeys had some form of dental disease i.e. missing teeth or at least one dental abnormality (shear, wave, step, undershot, overshot or diastema). Donkeys with dental disease (n = 1284), were significantly older than those without dental disease (n = 1635), (P < 0.001).

### Case-control study results

A total of 193 cases of impaction colic occurred during the case-control study period between January 1^st ^2003 and March 31^st ^2005. More than half (58%) of the impactions resulted in death or euthanasia. The majority of impactions occurred in the pelvic flexure (45%), although the site of 32% of the impactions was not identified.

Variables that were associated with the outcome (p < 0.2) in the univariable analysis and were considered for inclusion in the multivariable model were: age, weight, farm, extra rations, dental disease, previous colic, the routine treatments – flu vaccination, delousing, farriery, the medical examinations for opthalmalogical and musculo-skeletal problems. There were 10 farms included in our analysis, however due to small sample sizes at 4 of these farms they were regrouped into 7 farms (one group made up of those farms where the sample size was small). Univariable analysis indicated that one farm was significantly associated with colic and in the multivariable analysis this farm was compared to all others grouped together.

It was not possible to include the variables weight and dentition in the same model due to missing data in both of these categories. The weight of 139 cases and 305 controls were available for analysis and only 90 cases and 188 control animals had a dental examination 6 months prior to the colic. Therefore two multivariable models were developed.

Model 1a excludes dental disease (table 4) and uses 116 matched sets for analysis. It indicates that donkeys that are older and those that are lighter in weight were more likely to suffer impaction colic. The odds of suffering from colic were seven times higher for donkeys that previously had colic than those that had not suffered from colic before. In addition, previous musculo-skeletal problems increased the risk of colic and those fed extra rations were more likely to suffer impaction than those that weren't provided with extra feed. One particular farm also tended to increase the risk of colic.

Model 1a excluded 77 matched cases due to missing data in the weight category. When the backwards stepwise regression was repeated excluding weight, all other variables remained in the final multivariable model (model 1b, table 5). The magnitude of the odds ratio and significance of each variable (all P values <0.001) was strengthened by the inclusion of more matched sets.

Model 2 (table 6) which excludes weight and includes dental disease, uses 56 matched sets. Model 2 indicates that, even after adjusting for age, the presence of dental disease was significantly associated with impaction colic – donkeys with dental disease were more likely to suffer impaction colic than those without dental disease.

Examination of the delta betas and fitted values showed model 1 to be stable. Removal of the data points with the largest delta betas had little effect on either the variables included in the model, the magnitude of the odds ratios or the confidence intervals. Removal of the two data points with the largest delta betas in model 2 prevented the model from converging after 30 iterations. However, these were a control with dental disease and a case without dental disease, hence their removal would have the influence of increasing the effect of dental disease.

## Discussion

Our study identified all cases of colic in a large population of donkeys during a five year period. The incidence rate of colic in this population is similar to reports of incidence of colic in horses which range from 3.5–10.6 episodes per 100 horses per year [6,7]. The proportion of impactions diagnosed in this donkey population is much higher than the 5–12% that has been reported for horses [8,9]. However in a study of geriatric equidae, which were more than 20 years old, a higher frequency of large colon impaction was reported, (14.4% of colic cases) [10]. A proportion (37%) of the impactions in this donkey population were not confirmed by rectal palpation or post mortem and so were based on clinical signs only. This may have led to some misclassification of other types of colic or other medical conditions as impaction colics. However, this may be expected to dilute the effect of any risk factors towards the null.

The case fatality rate of 51% is considerably higher than that reported in horses, which varies from 6.7% – 15.6% depending on the population and colic type [7,11,12]. It is possible that this could be an overestimate of the mortality risk for two reasons. Firstly, some donkeys may have experienced a mild case of colic from which they recovered without it being identified or treated. Secondly, this mortality risk included cases of colic that were only confirmed at post mortem (n = 159). However, if these cases (n = 159) were excluded the case fatality rate would be 39.0% (253 deaths/648 colic cases) – a rate that is still much greater than that recorded in other equine populations. The mortality risk of impaction colic that we recorded in this donkey population was also high (51%) in comparison to other equids. For example, in a study of large colon impaction in horses, 95% of those that were treated medically and 58% of those treated surgically survived at least one year after the colic [13]. There are a number of reasons that might explain this difference. The age of the donkeys is likely to have a strong influence since this is a population of aged donkeys (mean age of 25 years) and those that did not survive were significantly older that those that recovered from the colic. A comparable study of aging equids (including less than 1% donkeys), which classed animals that were more than 20 years old to be geriatric (maximum age of 44 years), also recorded high mortality. They found that 47% of the colics (n = 89/191) resulted in death or euthanasia [10,14], however this was in a referred population where the severity of disease may be expected to be greater.

In our study the majority of donkeys that did not survive were euthanized, and it is likely that the decision for euthanasia also considered the health of other body systems. It is also possible that complications particularly associated with donkeys, such as hyperlipaemia, may have contributed to the higher case fatality rate. The case fatality rate in our study may also be high because surgery was rarely performed on the donkeys at the Donkey Sanctuary. In comparison surgery is performed more frequently on horses with an estimated 7% of colic cases requiring surgical management [8], although the number of surgical colics in older equids (more than 15 years old) may be significantly greater [8,15]. One study of a group of equids that were more than 20 years old noted that 36% of colics were treated surgically, of which 50% survived [10].

The duration of colic was longer than that in horses, where single episodes have been recorded to last for hours rather than days. For example, studies have noted that few horses (19%) showed colic signs for more than 12 hours [8], while others have recorded a maximum of 24 hours [16]. However large colon impactions tend to be of longer duration. A study of 147 cases of large colon impaction in horses found that the mean duration of abdominal pain prior to referral was 32 hours and the duration of medical treatment required to solve the impaction was a mean of 2 days (range 1 to 6 days) [13]. The duration of colic that we recorded may also be related to diagnosis and treatment. Surgery is commonly used to solve colic episodes promptly in horses; however it was rarely used to manage colic in donkeys in this population.

Diagnosis of colic in donkeys can be more difficult than in horses because donkeys show few overt signs of abdominal pain and colic may not be identified until the donkey is in the terminal stages of the disease [3]. In horses, protracted clinical signs without treatment have a worse prognosis due to the greater likelihood of dehydration and necrosis or tearing of the intestinal wall [17,18]. The presenting clinical signs and treatment of impaction colic in donkeys, the treatments received and their relationship to the outcome of colic (recovered or death/euthanasia) is the subject of ongoing research.

Our study demonstrated a seasonal pattern in the occurrence of impaction colic with peaks in autumn and troughs in the spring and early summer months. This result is consistent with a previous observation at the sanctuary, based on 12 months of data, that there was a higher monthly prevalence of colic in donkeys in October [2,3]. The peak in impaction colics in the autumn coincides with the change to winter housing which is associated with a change in diet and a decrease in exercise. These have been identified as risk factors for impaction colic [13,19-24]. The incidence of colic may also be seasonal in some horse populations and for some types of colic [6,8,11,16,25]. The timing of prevalence peaks vary between studies and the precise conditions predisposing this seasonal effect of colic are not well defined. It has been suggested that management factors related to certain times of year may be associated with colic, for example, changes in stabling, quantity of feed or amount of exercise [6,25]. One study noted a 12 month seasonal pattern in large colon impaction colic, where impactions peaked in the autumn and winter months and decreased in the spring with the lowest numbers in July and August [26]. These peaks coincided with times of management change or periods when horses were more likely to be intensively managed.

The retrospective case-control study identified several risk factors for impaction colic, although it was limited to variables that were routinely recorded in the database. Some of the variables that we identified as risk factors, in particular age, provision of concentrate feed and previous colic, have also been identified as risks in other equids [21,23,27]. Although some studies of the association between age and colic have produced conflicting views, many researchers have found that older equines are at increased risk of suffering colic [7,15].

Previous colic is frequently reported as a risk factor for future colic [21,23,27]. In this population donkeys were more likely to suffer impaction colic if they had suffered from colic of any type in the previous year. Other studies have noted similar results, for example, in one colic study, 43.5% of horses suffering from colic had suffered colic previously, 11% within one year [16]. Another study recorded a higher prevalence of colic than expected (32%) in horses following a case of large colon impaction [13]. The authors speculated that either colon dysfunction caused the original impaction and subsequently predisposed these horses to additional colic episodes, or that some permanent damage to the colon as a result of the impaction, was responsible. Others have also suggested that some horses are predisposed to colic due to a reduction in the density of neurons in the large colon and a thickening of caecal muscle which may increase the risk of recurrent colic from colon dysfunction [28,29].

Feed types have been identified as a cause of colic in equines, although the concentrate type, quantity and frequency of feeding require further investigation [4]. The donkeys in this study were given extra rations for a number of reasons; often concentrates were provided to underweight donkeys to improve their body condition or were given to donkeys with poor dentition, that as a result had difficulty eating forage. Although some studies in horses have found no association between colic and the type of concentrate [16,21], others have recorded that the amount of concentrate is a significant risk factor for colic [23,24]. One such study that tested increasing amounts of concentrate found that the odds of suffering from impaction colic were 6 times greater for horses being fed the greatest amount than for those that didn't receive any [23]. Importantly, a recent change in type or amount of concentrate fed is associated with increased risk [21-24]. For example one study noted that risk of colon obstruction increased in the 14 days after an increase in concentrate feeding [20]. Although weight is a significant risk factor for impaction colic in our model, we can conclude little without knowing the height or the body condition score of the animals, variables that were not recorded in the database.

Other factors i.e. farm, dental disease and musculo-skeletal problems are mentioned less frequently in the equine literature and are currently the subject of further investigation in this donkey population. Several possibilities exist for the apparent association between musculo-skeletal problems and suffering impaction colic. We hypothesize that donkeys with musculo-skeletal problems may have reduced their amount of exercise, may have more difficulty walking to water sources and may be confined or are less able to access pasture. These changes could contribute to the development of impaction colic. Confinement or reduced ability to access pasture could result in feeding changes which interfere with normal intestinal motility as has been suggested in horses [24]. Adequate exercise may also be important in maintaining normal large intestine function. In a comparison of resting and exercised donkeys, digestibility of dry matter, crude protein and cellulose increased in exercised donkeys, although the effect was not significant [30]. However, a significant increase in digestibility of feed as a result of exercise has been reported in horses [19]. Probably more important are recent changes in the amount of regular exercise. In a study of 147 horses, that suffered large colon impaction, 79 experienced a change in routine within the 2 weeks prior to developing the impaction [13]. Of these 47 (54%) horses experienced exercise restriction by enforced stall confinement because of a musculo-skeletal injury. The remaining 46% were restricted to stalls following arthroscopic surgery or were hospitalised for reasons other than gastro-intestinal disease. Similarly, another study noted that the development of colonic obstruction in horses was associated with a decrease in amount of exercise and increased number of hours stabled in the previous two weeks [20]. Finally musculo-skeletal problems may affect a donkey's ability to access water frequently and studies have shown that water deprivation is associated with an increased risk of large colon impaction [31]. In studies about colic in general, horses that did not have a continuous supply of water outdoors were more than twice as likely to suffer colic than those with adequate water [27], while horses with additional water sources such as ponds as well as buckets, troughs or tanks were at decreased risk [7,22].

Our study suggests that there is a relationship between colic and dental disease since we found an increased frequency of dental disease in donkeys that died or were euthanized due to colic than in those that survived. Furthermore, the case-control study indicated that dental disease was significantly associated with impaction colic. A previous descriptive study in the same donkey population also suggested a link between dentition and colic because dental disease was present in 54% of the impacted donkeys, however no control animals were evaluated [2,3]. A more recent study of an aged population of equidae (20 years or older), which only included a very small number of donkeys (less than 1%) also suggested a link. The authors noted that severe premolar and molar arcade abnormalities were often found in the animals with large colon impaction, although no control animals were evaluated [10,14]. Other studies on younger horses have also found a relationship, specifically poor dentition [31] or infrequent dental treatment [20] have been associated with large colon impaction or large colon obstruction respectively.

It has been suggested that dental disease can adversely affect the ability to adequately masticate feed for subsequent digestion and may result in long fibres entering the large colon, predisposing animals to intestinal tract obstruction [10]. These observations highlight the need for regular dental examinations. Indeed it has been suggested that most geriatric horses have some degree of dental disease including wave mouth, step mouth, excessive tooth wear and periodontal disease [14]. Although these authors found that 8% of the geriatric horses in their study population had dental disease, they suggest that the actual number with dental disease is likely to be much higher than determined from the records. This is because a thorough examination of the mouth would not have been performed on horses if they were admitted for a problem unrelated to the oral cavity.

In our study the farm was significantly associated with the occurrence of impaction colic. Of the ten farms included in the analysis one farm in particular was an increased risk for impaction colic. Farms differ in many ways, for example in management practices, number of care givers, feeding, housing and size of pasture. Identifying a specific cause is the subject of a prospective study.

## Conclusion

The results of this study confirm that colic affects the health and welfare in this aging population of donkeys in the south west of the UK and that both the incidence rate and the mortality risk are high compared to other equine populations. It reveals several risk factors for impaction colic some of which might be manipulated with the aim of reducing incidence of the disease, e.g.provision of concentrate feed or seasonal change in housing or access to pasture. It also highlights the importance of regular dental examinations as a preventive measure. Further prospective research aims to collect more detailed information about the risk factors for impaction colic in this population. For example, body condition scores, more detailed dental records or additional details of differences in farms may help us to assess the importance of risk factors that we were unable to explain in this retrospective analysis. In addition, further research about the disease in a large population of donkeys housed at private locations across the UK aims to determine the prevalence and risk factors for colic in a younger population of animals.

## Methods

This study used a retrospective analysis of a database designed and maintained by the Donkey Sanctuary. This database included identification, clinical and management records of all donkeys housed at ten farms owned and managed by the Donkey Sanctuary. Mules and ponies were excluded and donkeys not housed on sanctuary premises were also excluded.

### Identification of cases

Records of all donkeys that had been entered into the database from 1^st ^January 2000 to 31^st ^March 2005 were reviewed in order to identify all cases of colic that occurred during this time. Cases of colic were defined as any donkey showing clinical signs of colic as diagnosed by one of the resident veterinarians at the Donkey Sanctuary. These cases were identified by searching a specific coded column for all cases of colic categorised according to the veterinary diagnosis as surgical, impaction, spasmodic or undefined. In addition, to identify any cases entered under other categories, a search for text words was performed. This searched for all words and abbreviated words associated with the colic in transcribed text entered by the veterinarian at the time of examination (e.g. colic, abdomen, impacted, impaction). In addition all post mortem results were searched in a similar way to identify donkeys that died or were euthanized due to colic and to confirm diagnosis. A new episode of colic was defined as one when the donkey had been free from colic for the previous 14 days.

Impaction colic cases were diagnosed as the cause of colic either by rectal examination, post mortem examination or by the presenting clinical signs; mild to moderate colic, reduced faecal output, dried faecal material, and elimination of other causes of colic. The site of impaction colic was classified according to the following categories: pelvic flexure, large colon, small colon or rectum, caecum, small intestine or unidentified.

Information about each donkey that suffered colic was noted including age, gender, farm and the presence or absence of dental disease (at least one of the following features: missing teeth, shear, wave, step, undershot, overshot or diastema). Duration of colic was measured from the first examination until the date that the donkey recovered. When the exact date was not specified we recorded the last date on which the colic was mentioned and therefore recorded the minimum length of that colic episode.

### Retrospective matched case-control study

A retrospective matched case-control study was conducted using all cases of impaction colic recorded in the Donkey Sanctuary database between January 1^st ^2003 and March 31^st ^2005. We restricted the analysis to this time period because accurate dental records by a full time equine dental technician began in 2003. Each case was matched with two control animals that were housed at the sanctuary in the same month. Cases of impaction colic were identified as described above. Controls were defined as any donkey housed at the sanctuary during the same month as the case animal and which had not had colic in the previous 14 days. In this way the controls were matched on time. To select controls all donkeys on the premises in the month of the colic were ordered according to the individual donkey identification number. Two controls were selected for each case using random number generation in Microsoft Excel (Microsoft Office Excel 2003, Microsoft Corporation, USA). Information about each case and control was extracted from the database. Potential risk factors that were examined are shown in table 7.

### Data analysis

The number and types of colic that occurred during the study period were summed. Incidence rate was calculated as the number of new episodes of colic per 100 donkeys per year at risk. Multiple episodes in one donkey were included in this calculation since the aim was to estimate incidence rate of colic events; a new episode was recorded when the donkey had been free from colic in the previous 14 days. Denominator data were obtained by summing the number of donkey years at risk over the 63 month period.

T-tests were used to compare the age of donkeys that died from colic with those that recovered and to compare the age of those with dental disease to those without dental disease. The variable age was normally distributed.

To examine any seasonality in the occurrence of colic, the proportion of impaction colic cases (number of new episodes/total number of donkeys at risk) in each of the 63 months under investigation were recorded. Our a priori hypothesis based on previous work [2], was that there would be a seasonal component to colic with an autumn peak. To highlight any seasonal effects the raw data were smoothed using a 3 month moving average [32]. To further explore seasonality we used a binomial response regression model. The model incorporated cycles at both 12 and 6 month frequencies with sin12 and cos12 sinusoidal components representing seasonality in the form of a cycle with 12-month frequency and sin6 and cos6 representing 6-month cycles.

Analysis of the case-control study used conditional logistic regression methods using maximum likelihood estimation for both univariable and multivariable analyses, which take into account the matched design. Variables with P < 0.2 in the univariable analysis were considered for inclusion in the multivariable model. A backward stepwise elimination approach was used, in which all variables were initially included and then variables with P > 0.1 were sequentially removed if there was no significant influence on the fit of the model which was assessed by the change in deviance. It was not possible to fit all data into one multivariable model due to missing data in the dental disease and weight categories, we therefore present two models. Model 1 includes all variables except dental disease. Model 2 was built using all variables that could be included with dental disease, i.e. age, extra rations and farm. Evidence of confounding effects was examined for all variables in the final models. Each potentially confounding variable was added one at a time to the final model and the effect on the remaining variable's status in the model was assessed. None of the variables had a significant effect on others in the model (the odds ratio of each variable did not change by more than 25% when the variable was included) and therefore were excluded from the model. Interaction terms were tested between all biologically plausible sets of terms by including interactions in the model one at a time to assess their effect on the fit of the model. Any interaction term where P > 0.1 was excluded from the final model. The fit and stability the model was assessed by examining the delta-betas [33].

Conditional logistic regression analysis was conducted in EGRET (Egret for Windows 2.0, Cytel Software Corporation, 1999). All other analyses were conducted in SPSS version 12 (SPSS Inc 2003).

## Authors' contributions

RC participated in design of the study, carried out descriptive and statistical analysis and drafted the manuscript. CJP provided guidance and advice on aspects of analysis and writing. AFT oversaw project design and approved the final manuscript. FB managed and developed the use of the database and contributed to interpretation of data. GLP conceived of the study, participated in design and analysis and provided guidance in manuscript writing.

## Figures and Tables

**Table 1:** Colic episodes in a population of 4596 donkeys housed at the Donkey Sanctuary between January 2000 and March 2005.

	**Recovered from colic**	**Died/euthanized following colic**	**Total**
Impaction colic *(including impactions requiring surgery)*	215	227	442
Other colic *(including surgical, spasmodic and undiagnosed)*	180	185	365
**Total**	**395**	**412**	**807**

**Table 2:** Site of impaction in donkeys diagnosed with impaction colic (n = 442) between January 2000 and March 2005.

**Impaction site**	**Recovered from impaction**	**Impaction resulted in death or euthanasia**	**Total impactions**
Pelvic flexure	79	96	175
Large colon	2	31	33
Small colon	10	20	30
Caecum	16	21	37
Small intestine	0	3	3
Unidentified	108	56	164
**Total**	**215**	**227**	**442**

**Table 3:** Parameter estimates from the regression model with a seasonal component corresponding to a 12 month cycle, for proportion of impaction colics per month between January 2000 and March 2005.

	**Parameter estimate**	**Lower 95% CI**	**Upper 95% CI**	**P-value**
Intercept	-5.92	-6.02	-5.83	
Sin 12	-0.08	-0.22	0.05	0.2
Cos 12	0.19	0.05	0.32	0.006

**Table 4:** Multivariable conditional logistic regression model 1a of risk factors for impaction colic in donkeys housed at the Donkey Sanctuary between 1st January 2003 and 31st March 2005.

**Variable**		**Odds Ratio**	**Lower 95% CI**	**Upper 95% CI**	**LRS P-value**
**Continuous**					

Age (years)		1.06	1.02	1.11	0.008
Weight (kg)		0.98	0.96	0.99	<0.001

**Categorical**					

Extra rations	No	1.00			
	Yes	2.21	1.07	4.53	0.03
Musculo skeletal exam	No	1.00			
	Yes	2.20	0.95	5.13	0.07
Previous colic	No	1.00			
	Yes	6.80	1.65	27.95	0.008
Farm	1	1.00			
	2	0.43	0.19	0.97	0.04

Model 1a excluded the variable dental disease during the backwards stepwise regression. LRS = likelihood ratio test statistic

**Table 5:** Multivariable conditional logistic regression model 1b of risk factors for impaction colic in donkeys housed at the Donkey Sanctuary between 1st January 2003 and 31st March 2005.

**Variable**		**Odds Ratio**	**Lower 95% CI**	**Upper 95% CI**	**LRS P-value**
**Continuous**					

Age (years)		1.09	1.05	1.12	<0.001

**Categorical**					

Extra rations	No	1.00			
	Yes	2.48	1.49	4.12	<0.001
Musculo skeletal exam	No	1.00			
	Yes	2.60	1.36	4.99	0.004
Previous colic	No	1.00			
	Yes	8.81	2.66	29.2	<0.001
Farm	1	1.00			
	2	0.37	0.21	0.64	<0.001

Model 1b excluded the variables dental disease and weight during the backwards stepwise regression. LRS = likelihood ratio test statistic

**Table 6:** Multivariable conditional logistic regression model 2 of risk factors for impaction colic in donkeys housed at the Donkey Sanctuary between 1st January 2003 and 31st March 2005.

**Variable**		**Odds Ratio**	**Lower 95% CI**	**Upper 95% CI**	**LRS P-value**
**Continuous**					

Age (years)		1.09	1.00	1.182	0.03

**Categorical**					

Dental disease	No	1.00			
	Yes	29.73	3.95	223.67	<0.001

Model 2 included the variables age, dental disease' farm and extra rations during the backwards stepwise regression. LRS = likelihood ratio test statistic

**Table 7:** Variables included in the matched case-control univariable analysis.

**Variable**	**Description**
Age	Years
Gender	Male/female
Weight	Kg
Farm	10 different farms in the south west of England were included in the analysis.
Extra rations	Donkey was fed concentrate feed in addition to forage at the time of the impaction colic.
Previous colic	Donkey suffered from colic in the 12 months prior to the impaction colic case.
Dental disease	Donkey had at least one of the following dental problems recorded in the 6 months prior to the impaction colic date: missing teeth, shear, wave, step, undershot, overshot or diastema.
Routine treatments: *farriery, vaccination, antiparasiticide, anthelmintic*.	Donkey had routine treatment in the month prior to impaction colic case date. Each category was tested separately in the univariable analysis.
Medical examinations: *behavioural, biochemical, dermatalogical, digestive, haematological, musculo-skeletal, non-specific, opthalmalogical, respiratory, uro-genital*.	Donkey had a medical examination for another disease in the 6 months prior to the impaction colic case date. Medical examinations were categorised by Donkey Sanctuary veterinarians. Each category was tested separately in the univariable analysis.
